# Dermoscopic Features of Eccrine Porocarcinoma Arising from Hidroacanthoma Simplex

**DOI:** 10.1155/2010/192371

**Published:** 2010-10-11

**Authors:** Reiko Suzaki, Takeaki Shioda, Izumi Konohana, Sumiko Ishizaki, Mizuki Sawada, Masaru Tanaka

**Affiliations:** ^1^Department of Dermatology, Tokyo Women's Medical University Medical Center East, 2-1-10 Nishi-Ogu, Arakawa-ku, Tokyo 116-8567, Japan; ^2^Department of Dermatology, Hiratsuka City Hospital, 1-19-1, Minamihara, Hiratsuka city, Kanagawa 254-0065, Japan

## Abstract

Eccrine porocarcinoma is a rare cutaneous neoplasm that mainly affects elderly people and grows slowly over a long period of time but often experiences an accelerated growth phase. Eccrine porocarcinoma may arise de novo or evolve from a pre-existing benign eccrine poroma. We reported a 86-year-old Japanese woman presenting with two reddish-colored pendulated lesions on a keratotic light brown plaque on the right thigh. Dermoscopic examination of the light-brown plaque demonstrated many whitish globular structures in a light-brown background. At the two reddish-colored pendulated lesions, polymorphous and prominent vessel proliferation was observed together with irregularly shaped whitish negative network. Immunohistochemical study demonstrated a positive CEA staining at ductal structures and atypical clear cells of reddish nodules. A diagnosis of eccrine porocarcinoma arising in a pigmented hidroacanthoma simplex was eventually established, and the dermoscopic features of eccrine porocarcinoma from hidroacanthoma simplex was described for the first time.

## 1. Case Report

**Figure 1:**
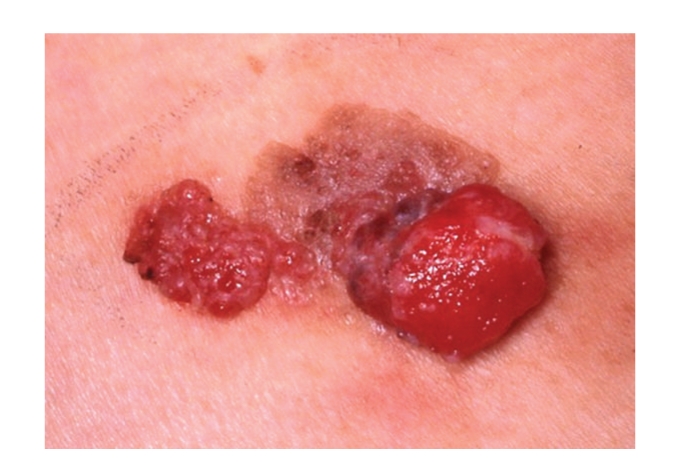
Two reddish-colored pendulated lesions at the peripheries of a keratotic light brown plaque.

**Figure 2:**
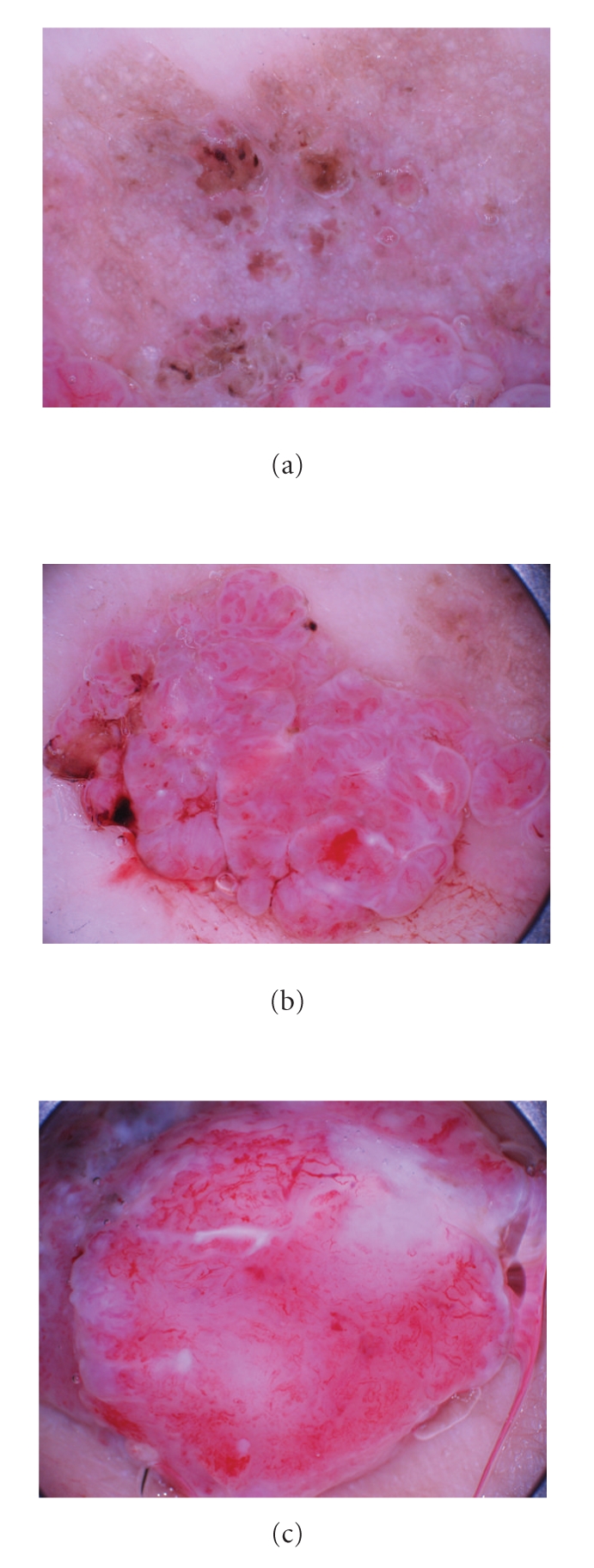
(a) Dermoscopic examination of the light brown plaque demonstrated a lot of whitish globular structures on the light brown background. (b) In the left pendulated nodule, prominent polymorphous vessels were observed together with irregularly shaped whitish negative network. (c) Vessels were more conspicuous and irregular in the right nodule.

A 86-year-old Japanese woman presented at Hiratsuka City Hospital with a cutaneous tumor on her right thigh. Examination revealed two reddish-colored pendulated lesions at the peripheries of a keratotic light brown plaque of 27×17 mm (Figure 1), which had been noticed for 5 years. Half a year ago, two reddish-colored nodules began to grow slowly on this plaque. There was no lymphadenopathy. Dermoscopic examination of the light brown plaque demonstrated many whitish globular structures on the light brown background. There were a few comedo-like, well-circumscribed dark brown structures surrounded by yellowish pink erosive areas (Figure 2(a)). At the two reddish-colored pendulated lesions, prominent polymorphous vessels, such as dotted, linear-irregular, glomerular, hairpin vessels were observed together with irregularly shaped whitish negative network (Figures 2(b) and 2(c)). Vessels were more conspicuous and irregular in the right nodule (Figure 2(c)). We clinically suspected these lesions as squamous cell carcinoma arising from seborrheic keratosis and performed an excision with a 1-cm margin. 

**Figure 3:**
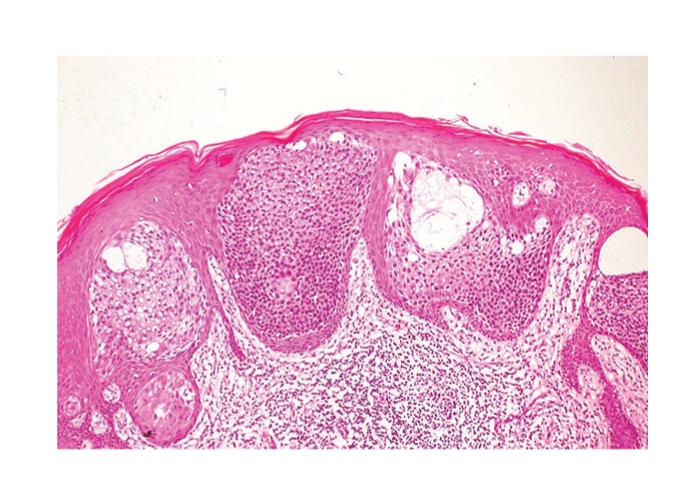
Histopathologic examination of the flat pigmented plaque disclosed many well-defined nests within the epidermis. (HE ×200).

**Figure 4:**
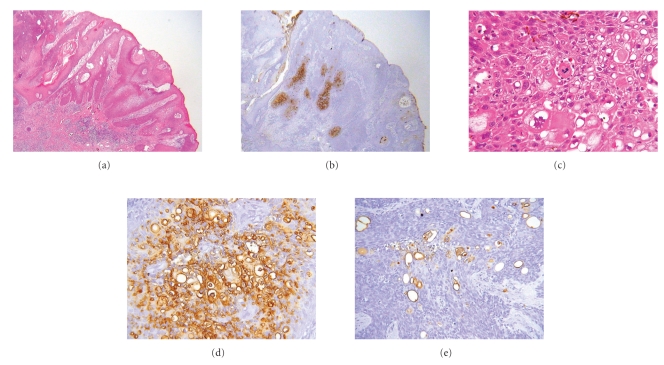
(a) In the right red nodule, the epidermis was prominently acanthotic with intraepidermal proliferation of clear cells and squamoid cells. Dermal invasion of atypical squamoid cells was partially apparent (HE ×20). (b) In the right red nodule, the tumor cells were focally positive for carcinoembryonic antigen (CEA, ×20). (c) In the right red nodule, markedly atypical cells proliferated and extended throughout the entire thickness of the acanthotic epidermis (HE ×400). (d) The atypical clear cells expressed CEA (CEA ×400). (e) The ductal structures expressed CEA (CEA, ×400).

**Figure 5:**
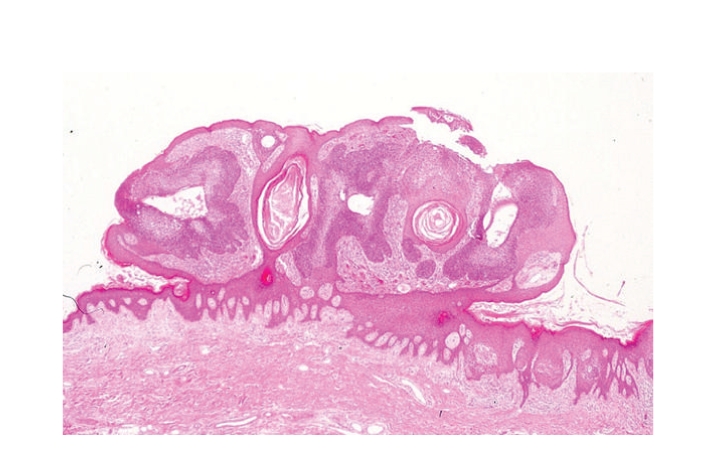
In the left red nodule, although the atypical basaloid cells proliferated in the epidermis, there were no visible invasions in the dermis (HE ×40).

Histopathologic examination of the flat pigmented plaque disclosed many well-defined nests within the epidermis (Figure 3). The nests were composed of cuboidal to oval or occasionally elongated, bland, basaloid cells. These lesions represented intraepidermal epithelioma of Borst-Jadassohn with mild cytological atypism and conformed to the histopathologic features of hidroacanthoma simplex or clonal type of Bowen's disease. In the right red nodule, markedly atypical cells proliferated and extended throughout the entire thickness of the acanthotic epidermis with apparent dermal invasion (Figures 4(a) and 4(c)). There were small ductal structures with cuticular layer in the tumor. The ductal structures and atypical clear cells expressed carcinoembryonic antigen (CEA) (Figures 4(b), 4(d), and 4(e)). Therefore, we considered this nodule as eccrine porocarcinoma. In the left red nodule, although the atypical basaloid cells proliferated in the epidermis, there was no visible invasion in the dermis (Figure 5). We considered this lesion as eccrine porocarcinoma in situ. The invasive part of eccrine porocarcinoma intensely expressed tumor protein p53 positive, but other areas were negative with p53.

Since the entire tumors were continuous, we made a final diagnosis of eccrine porocarcinoma and eccrine porocarcinoma in situ arising from pre-existing lightly pigmented hidroacanthoma simplex. 

The patient had been doing well after the operation. However, 8 months later, a right inguinal lymph node metastasis was found. After the inguinal lymph node dissection was performed, lung and para-aortic lymph node metastases were confirmed on the CT. The patient's general condition deteriorated and died 16 months after the surgery.

## 2. Discussion

Hidroacanthoma simplex (HAS) is a benign eccrine tumor that is also known as intraepidermal poroma. Eccrine porocarcinoma (EPC) is a rare cutaneous neoplasm that grows slowly over a long period of time but often experiences an accelerated growth phase [1]. EPC may arise de novo or evolve from a pre-existing benign eccrine poroma. We report an unusual case of EPC arising from pigmented HAS, and the dermoscopic features of EPC from HAS was described for the first time. 

Dermoscopy improves the clinical diagnosis of many pigmented and nonpigmented skin tumors including eccrine poroma. We described additional dermoscopic features for eccrine porocarcinoma and hidroacanthoma simplex. 

Only a few studies were published about the dermoscopic features of eccrine poroma. Altamura et al. published a case of nonpigmented eccrine poroma of the pubic region that simulated amelanotic melanoma, both clinically and dermoscopically. They described pink to reddish, irregularly shaped and sized structures reminiscent of milky-red areas, red lacunes, and linear irregular vessels [2]. Nicolino et al. described two cases of eccrine poroma that displayed two different patterns: one case featured a blue-white colour with an eccentric black blotch and hairpin vessels whereas the second one was characterized by a polymorphous vascular pattern composed of red lacunes, glomerular and linear vessels surrounded by a halo, pink-to-white in colour [3]. Kuo and Ohara described, for the first time, two cases of pigmented eccrine poroma with dermoscopic features that mimicked those of pigmented basal cell carcinoma, such as blue-gray ovoid nests, multiple blue-gray dots, and arborizing vessels [4]. Ferrari et al. described seven cases of eccrine poroma, and three dermoscopic “profiles” were identified, all characterized by the presence of a white-to-pink halo surrounding the vessels, as well as by the association of two additional different combination of features, namely, a combination of glomerular vessels and pink-white structureless areas, a combination of glomerular and linear irregular vessels, and a combination of hairpin vessels and linear irregular vessels [5].

The combination of atypical vascular pattern and milky-red globules is especially specific for the diagnosis of eccrine porocarcinoma. The former is also observed in other lesions, such as amelanotic melanoma, actinic keratosis, Bowen's disease, and basal cell carcinoma and squamous cell carcinoma.

In this paper, we have shown characteristic dermoscopic features of a lot of whitish globular structures in the light brown back ground. Each whitish round structure would correspond to a nest of cellular proliferation of hidroacanthoma simplex in the epidermis. Since the nests of poroma cells are entirely in the epidemis, hidroacanthoma simplex would only show whitish globular structures without vessels. There are comedo-like, well-circumscribed dark brown structures, which would correspond to keratotic pluggings in the surface. In the two reddish-colored lesions, polymorphous and prominent vessel proliferation is observed together with irregular whitish negative network. Vessels included dotted, linear-irregular, glomerular, and hairpin. Vessels were more conspicuous and irregular in the EPC. The present case is composed of EPC and EPC in situ on the plaque of HAS, and the obvious difference in vessels on dermoscopy could be a helpful clue for an estimation of the grade of malignancy.

Although it would be difficult to differentiate amelanotic melanoma from EPC on dermoscopy alone, the peripheral findings suggesting HAS, such as whitish globular structures on the homogeneous brown area, might be helpful for a diagnosis of EPC.

In this case, although the three portions of the tumor were continuous, the pathological difference well explained the difference in dermoscopic features. Evaluations of various skin lesions using dermoscopy would be indispensable in the daily dermatology practice.
